# Study on risk factors and treatment strategies of hypoxemia after acute type a aortic dissection surgery

**DOI:** 10.1186/s13019-024-02775-y

**Published:** 2024-05-03

**Authors:** Wenbo Yu, Yuan Liang, Jianfeng Gao, Jianxian Xiong

**Affiliations:** 1 The First Clinical Medical College of Gannan Medical University, Ganzhou, 341000 China; 2 First Affiliated Hospital of Gannan Medical University, Ganzhou, 341000 China

**Keywords:** Acute type a aortic dissection, Hypoxemia, Risk factors, Treatment strategy

## Abstract

Acute type A aortic dissection is a life-threatening cardiovascular disease characterized by rapid onset and high mortality. Emergency surgery is the preferred and reliable treatment option. However, postoperative complications significantly impact patient prognosis. Hypoxemia, a common complication, poses challenges in clinical treatment, negatively affecting patient outcomes and increasing the risk of mortality. Therefore, it is crucial to study and comprehend the risk factors and treatment strategies for hypoxemia following acute type A aortic dissection to facilitate early intervention.

## Introduction

Acute type A aortic dissection (ATAAD) is a severe cardiovascular disease with significant disability and mortality [1]. The patient’s condition rapidly deteriorates, with the mortality rate increasing by 1% within the first hour of onset. Without prompt medical intervention, the mortality rate can reach as high as 30-50% within 48 h [2]. In China, Sun’s surgery is the primary surgical approach for treating ATAAD. However, this operation is challenging, time-consuming, highly complex, and invasive. Additionally, aortic arch replacement must be performed under deep hypothermic circulatory arrest (DHCA) and selective cerebral perfusion [3]. During circulatory arrest, vital organs such as the heart, lungs, spinal cord, kidneys, liver, and gastrointestinal tract experience ischemia and hypoxia, which can lead to reversible or irreversible organ malperfusion syndrome [4].

Hypoxemia (HO) is a common complication following ATAAD surgery [5], with an incidence rate as high as 51%. It can lead to acute lung injury and affect disease recovery [6]. Postoperative hypoxemia is caused by a combination of factors including aortic tissue destruction, ischemia/reperfusion injury, intraoperative cardiopulmonary bypass (CPB), DHCA, and massive blood transfusion [7]. These factors contribute to systemic and local inflammatory responses. The consequences of hypoxemia include an increased risk of prolonged ventilation time, ventilator-related lung injury, and nosocomial infection. In severe cases, the patient’s life may be endangered [8, 9]. The 30-day mortality rate stands at 6.3% [10], while the long-term mortality rate ranges from 21 to 50% [11, 12].

The purpose of this article is to provide an overview of the risk factors and treatment strategies associated with hypoxemia following surgery for ATAAD.

## Diagnostic criteria for hypoxemia

Hypoxemia refers to a condition where there is an insufficient amount of oxygen in the blood, resulting in a lower-than-normal partial pressure of arterial oxygen (PaO2) for individuals of the same age. This is caused by damage to the alveolar epithelial and capillary endothelial cells, leading to a decrease in blood oxygen partial pressure and saturation. The severity of hypoxemia is often evaluated using the oxygenation index (PaO2/FiO2), with a value of ≤ 300 mmHg indicating hypoxemia [13]. There are two types of hypoxemia: isolated hypoxemia and acute respiratory distress syndrome [10]. The normal range for arterial blood oxygen partial pressure is 90–100 mmHg, and hypoxemia is diagnosed when it falls below 60 mmHg. The Berlin definition classifies hypoxemia into three categories based on its severity: mild (PaO2/FiO2 range of 200–300 mmHg), moderate (range of 100–200 mmHg), and severe (PaO2/FiO2 < 100 mmHg) hypoxemia [14].

## Risk factors of hypoxemia

### Patients factors

#### Smoking history

Chronic smoking has been found to cause pathological damage to lung tissue and trigger cigarette smoke-induced airway inflammation and remodeling. This process involves the expansion of alveolar air space and the remodeling of small airways, which ultimately leads to airflow obstruction [15]. Following exposure to smoke, epithelial cells are the first to undergo changes and play a crucial role in subsequent inflammatory responses. Macrophages also play a significant role in the inflammation process by producing proteolytic enzymes that can damage lung tissue, resulting in respiratory dysfunction. Several studies have reported that a history of smoking is an independent predictor of postoperative hypoxemia in various surgical procedures [16–18].

#### Obesity

Obesity, defined as a BMI greater than 25 kg/m2, has a significant negative impact on cardiopulmonary physiology [19]. Numerous studies have demonstrated that an increase in BMI is a risk factor for postoperative hypoxemia [20]. The accumulation of fat on the chest wall and abdomen in obese individuals restricts the movement of the diaphragm and chest wall, reduces chest wall compliance, increases respiratory resistance, and elevates pleural pressure. Obese patients commonly experience a notable decrease in lung compliance, leading to increased dyspnea and respiratory resistance [21]. Consequently, the risk of postoperative hypoxemia is heightened. In obese patients with aortic dissection, elevated malondialdehyde levels indicate an imbalance between oxidation and antioxidants, triggering the synthesis of more secretions and inflammatory factors [22], as well as activating inflammatory signaling pathways. This, in turn, contributes to the development of postoperative hypoxemia. A study by Ming Gong et al. [23] found a higher likelihood of postoperative hypoxemia in obese patients and identified obesity as a risk factor for severe postoperative hypoxemia after ATAAD surgery.

#### Inflammatory reaction

When Acute Type A Aortic Dissection (ATAAD) occurs, blood enters the middle layer through the ruptured intima, leading to the accumulation of blood in the aortic wall. This accumulation triggers an inflammatory response, which plays a significant role in the development of ATAAD. Over time, this local inflammation can progress to systemic inflammation. The activation of inflammatory substances in the lungs can cause damage to alveolar cells, affecting the integrity of lung tissue capillaries. Consequently, this leads to pulmonary capillary endothelial edema, abnormal ventilation-perfusion ratio, intrapulmonary shunting, and reduced pulmonary gas exchange [24]. These factors ultimately impact the patient’s oxygenation function, resulting in respiratory dysfunction. Surgery for ATAAD patients is associated with greater trauma, longer duration, extracorporeal circulation, and blood transfusion. These factors further exacerbate the inflammatory response in the patient’s lungs post-surgery, leading to hypoxemia. Research has shown that leukocytes are biomarkers that reflect systemic inflammatory response and are independently associated with postoperative hypoxemia [25, 26]. In recent years, clinicians have increasingly recognized the relationship between inflammatory response and postoperative hypoxemia [27]. The inflammatory factors caused by the inflammatory response can damage lung tissue and affect respiratory function, which is closely linked to postoperative hypoxemia in ATAAD patients.

### Intraoperative related factors

#### Cardiopulmonary bypass and deep hypothermic circulatory arrest

CPB is an essential component of ATAAD surgery [28] and is widely utilized in cardiac surgery [29]. CPB triggers a robust inflammatory response, leading to the production, release, and circulation of vasculogenic and cytotoxic substances. These inflammatory responses have been found to cause multiple organ dysfunction, including the lungs [30]. Acute lung injury is a significant complication in CPB surgery [31]. It is characterized by increased pulmonary capillary permeability, extravascular and interstitial edema, accumulation of alveolar fluid, elevated interstitial lung water, and changes in surface Reactive substances, resulting in decreased lung compliance and gas exchange function [32]. These changes collectively contribute to increased local atelectasis, heightened susceptibility to infection, and augmented physiological arteriovenous short circuiting, ultimately leading to decreased arterial oxygen saturation. DHCA is a specialized procedure in extracorporeal circulation that plays a crucial role in safeguarding brain tissue during ATAAD surgery and creating a relatively blood-free surgical area, thereby enhancing the likelihood of surgical success [33]. The use of moderate hypothermia (nasopharyngeal temperature 25–28 °C) during DHCA has been shown to have a lower risk of systemic complications and a reduced incidence of postoperative hypoxemia compared to deep hypothermia (18–20 °C) [34]. Research indicates that moderate hypothermic circulatory arrest with unilateral antegrade cerebral perfusion, as opposed to deep hypothermic circulatory arrest without cerebral perfusion, can lead to shorter ventilation times and decreased risk of hypoxemia [35]. During deep hypothermic circulatory arrest (DHCA), the lungs experience complete ischemia and hypoxia, remaining in a non-ventilated state for an extended period. This can result in the collapse of certain alveoli, leading to atelectasis and a reduction in lung volume. Consequently, there may be an imbalance in the ventilation/blood flow ratio, which can contribute to the occurrence or worsening of postoperative hypoxemia [36]. This process is primarily characterized by lung injury caused by ischemia/reperfusion of lung tissue [37]. Several studies have demonstrated that CPB and DHCA during surgery for acute type A aortic dissection (ATAAD) patients can exacerbate lung damage and increase the likelihood of postoperative hypoxemia. Liu et al. found that DHCA lasting longer than 25 min significantly raises the risk of postoperative hypoxemia [26]. Multiple studies have identified CPB/DHCA as a risk factor for postoperative hypoxemia in ATAAD patients [4, 19, 21, 23, 38].

#### Infusion of blood products during operation

Challenges faced during surgical procedures for patients with acute type A aortic dissection (ATAAD) include difficult surgical techniques, greater trauma, consumptive coagulopathy caused by blood flowing through the false cavity, and increased hemodilution, platelet activation, and aggregation caused by extracorporeal circulation. These factors can contribute to heavy bleeding and refractory excessive blood loss during surgery [39]. Therefore, it is often necessary to administer different blood products during or after surgery to improve blood circulation and prevent ischemia. Hypoxia can lead to damage in multiple organs [40], making blood transfusion inevitable for ATAAD surgery [41]. However, blood transfusion is also associated with a series of adverse reactions, including allergies, infections, immune reactions, transfusion-related organic damage, and circulatory overload. One severe adverse reaction to blood transfusion is transfusion-related acute lung injury (TRALI), characterized by acute onset of pulmonary permeability edema and impaired respiratory function within 6 hours after transfusion [42]. TRALI is considered a ‘double-hit syndrome’ in which activated neutrophils in the lung interact with endothelial cells, causing endothelial damage, vascular leakage, and pulmonary edema, ultimately leading to lung injury [43]. The administration of large amounts of blood products may result in associated lung injury, leading to postoperative hypoxemia [44]. Additionally, patients who receive transfusions have 3.4-fold increased odds of developing postoperative pneumonia, with the odds increasing significantly with each additional unit of red blood cells transfused [45].

#### Other factors

Age has been reported to be associated with the occurrence of hypoxemia during various surgeries [46]. Sheng et al.‘s study demonstrated that older age independently predicts early postoperative hypoxemia and confirmed that the incidence of postoperative hypoxemia significantly increases with age [18]. Furthermore, preoperative renal insufficiency is also identified as a risk factor for postoperative hypoxemia. Wang et al. investigated the relationship between renal insufficiency and respiratory complications [47, 48]. The inflammatory response and renal regulation of erythropoietin production, and consequently oxygen delivery, may play crucial roles in this association [49]. If malperfusion syndrome is present before surgery for ATAAD, the surgery may worsen organ malperfusion, extend the patient’s time in bed and stay in the Intensive Care Unit (ICU), increase the likelihood of pulmonary complications, and result in hypoxemia. Therefore, addressing organ malperfusion before surgery can help decrease the occurrence of postoperative hypoxemia. The management of preoperative ATAAD combined with organ malperfusion syndrome remains a contentious issue. In our country, emergency central aortic repair is commonly carried out to save the patient’s life and decrease mortality rates. However, this approach does not necessarily prevent postoperative hypoxemia in ATAAD patients with preoperative malperfusion syndrome.

## Treatment interventions for hypoxemia

### Prone position positive pressure mechanical ventilation

Mechanical ventilation is a crucial postoperative measure following ATAAD to enhance oxygen saturation and prevent atelectasis [50]. In a study by Xie et al., involving 384 patients post-ATAAD surgery, it was observed that mechanical ventilation aids in the rapid recovery of cardiopulmonary function and circulation following lengthy, traumatic, and complex surgeries. It is essential to extubate patients promptly to reduce the risk of mechanical ventilation-related complications. The study revealed a significant association between the administration of packed red blood cells, fresh frozen plasma, or platelet concentrates and the duration of postoperative mechanical ventilation. Among 213 cases, 55.47% required mechanical ventilation for over 24 h, 35.42% for over 48 h, and 25.00% for over 72 h [40]. Positive pressure mechanical ventilation plays a vital role in improving postoperative hypoxemia and saving patients’ lives [51]. The prone position (PP) can reduce pleural pressure and alter the vertical pleural pressure gradient, leading to a significant enhancement in lung mechanics. In a study by Katira et al., two lung injury models (normal saline lavage and large-volume ventilation) were used on 14 mechanically ventilated pigs. The researchers compared the effects of lung ventilation between supine and prone positions, revealing that the prone position resulted in longer ventilation of the head and tail of the lungs, ultimately improving ventilation efficiency [52]. . Additionally, PP can enhance gas exchange in damaged lungs, improve patient survival rates, shorten mechanical ventilation time and ICU stay, and serve as an effective treatment for acute respiratory failure [53, 54]. Previously, clinicians often utilized supine mechanical ventilation to address postoperative hypoxemia in ATAAD patients. Ding et al. conducted a study involving 96 patients with hypoxemia after ATAAD. The patients were divided into a control group (*n* = 48) and a treatment group (*n* = 48). The control group received traditional supine position ventilation treatment, while the treatment group received early prone position ventilation treatment. The results indicated that the improvement in lung function was significantly better in the treatment group compared to the control group [55], suggesting that prone positioning can elevate resting lung volume without altering the lung recruitment volume sustained by positive end-expiratory pressure [56]. Furthermore, PP can elevate transpulmonary pressure, enhance oxygenation and hemodynamics, and has proven effective in improving the prognosis of patients with acute respiratory distress syndrome (ARDS) and reducing ventilator-induced lung injury [57]. Therefore, the combination of positive pressure mechanical ventilation and prone positioning can be employed as part of a lung-protective ventilation strategy in patients with moderate to severe postoperative hypoxemia [58].

### Nasal high flow humidified oxygen therapy combined with nitric oxide inhalation

High-flow nasal oxygen therapy (HFNO) is a novel therapeutic approach currently employed in clinical practice. A study involving 120 high-risk patients for postoperative pulmonary complications compared the effects of HFNO therapy with traditional oxygen therapy. The results showed that after 24 h of oxygen therapy, patients in the HFNO group had higher oxygenation index, lower respiratory frequency, and reduced sputum viscosity grade. Additionally, the follow-up oxygen therapy time and postoperative hospitalization time were significantly shorter in the HFNO group. These findings suggest that HFNO can enhance respiratory function by decreasing anatomical dead space in the nasopharynx, leading to improved oxygenation [59]. HFNO can effectively prevent respiratory failure following extubation and obviate the need for reintubation by ensuring adequate oxygenation, promoting sputum excretion, and reducing the work of breathing [60]. In patients with ATAAD after Sun’s surgery, the administration of HFNO can mitigate the loss of lung volume and inspired oxygen concentration [61]. Nitric oxide (NO) is a selective pulmonary vasodilator that dilates the blood vessels supplying the alveoli, thereby increasing blood flow. This rapid improvement in ventilation/perfusion matching enhances oxygenation in the injured lung. In this study, 43 patients who experienced hypoxemia after surgery for ATAAD were treated with low-dose NO therapy. The results showed improvement in hypoxemia levels at 6, 24, 48, and 72 h after treatment initiation, as well as a reduction in the duration of ventilator support and ICU hospitalization [7]. In clinical practice, NO therapy is typically combined with nasal high-flow humidified oxygen therapy for patients with hypoxemia after ATAAD surgery. This combined approach significantly reduces the duration of invasive mechanical ventilation. Studies have demonstrated that NO treatment ameliorates postoperative hypoxemia by reducing intrapulmonary shunting [62]. The combination of nasal high-flow humidified oxygen therapy and nitric oxide inhalation therapy improves lung oxygenation capacity, reduces oxygen consumption, and promotes airway patency. This treatment strategy markedly enhances the prognosis of postoperative hypoxemia in ATAAD patients, leading to improved survival rates and accelerated recovery of the body.

### Drug intervention

Clinically, the treatment for patients with postoperative hypoxemia after ATAAD mainly involves mechanical ventilation with a ventilator and necessary drug-assisted treatment. Ulinastatin (UTI), a commonly used urinary trypsin inhibitor, is primarily used to treat acute or critical inflammatory reactions and organ failure. Medication can result in leukopenia or eosinophilia, as well as symptoms such as nausea, vomiting, and diarrhea. It may also cause elevations in AST and ALT levels, along with the possibility of severe allergic reactions. In their study, Jiang et al. divided 18 acute pulmonary edema rat animal models into 3 groups: control group, lipopolysaccharide (LPS) group, and LPS + UTI group. The findings indicated that UTI can upregulate TJ protein, Na-K-ATP enzymes, and ENaC to decrease permeability and increase alveolar fluid clearance, ultimately leading to improvement in pulmonary edema [63]. In the study conducted by Xu et al., a total of 36 patients with ATAAD were randomly assigned to receive either intraoperative administration of UTI (experimental group, *n* = 18) or 0.9% normal saline (control group, *n* = 18), as a result, UTI can be utilized to enhance the oxygenation function of the lungs after CPB, consequently improving postoperative hypoxemia in patients [64]. Fifty-five patients diagnosed with ATAAD were randomly assigned to two groups: the UTI group (*n* = 17) and the control group (*n* = 38). The study findings revealed that the incidence of postoperative hypoxemia was 23.5% in both the UTI and control groups, with an overall rate of 55.3%. Patients in the UTI group showed a significant improvement in postoperative hypoxemia compared to those in the control group, while no significant difference in mortality was observed between the two groups [9]. To mitigate the inflammatory response during CPB, prophylactic use of high-dose methylprednisolone can be considered, followed by low-dose treatment for 72 h after surgery to lower the incidence of lung injury and postoperative hypoxemia. In the study by Bai et al., it was found that after receiving prophylactic methylprednisolone treatment, there were a total of 34 cases of hypoxemia, resulting in an incidence rate of 27.4%. This incidence rate was significantly lower compared to patients who did not receive methylprednisolone treatment [44]. Therefore, when selecting drugs for patients with hypoxemia after ATAAD surgery, in addition to conventional antibiotics, the inclusion of low-dose methylprednisolone and UTI for 72 h after surgery can be considered to reduce lung damage and improve lung function and oxygenation.

### Extracorporeal membrane oxygenation

**Fig. 1:**
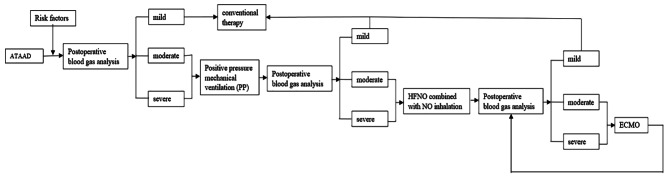
ATAAD postoperative hypoxemia diagnosis and treatment steps flow chart

When extracorporeal membrane oxygenation (ECMO) is utilized for the treatment of postoperative hypoxemia in ATAAD patients, it is primarily recommended for those with severe ARDS, in situations where conventional lung protective mechanical ventilation is unable to effectively prevent hypoxia or hypercapnia [65]. The 30-day hospital survival rate for patients with ATAAD and severe postoperative circulatory and respiratory dysfunction, who were treated with ECMO, was found to be 27.27% [66]. In cases where the location and hemodynamic status of the false lumen tear in the residual dissected aorta are uncertain, cannulae are typically inserted through the femoral artery and vein for veno-arterial (VA) ECMO. However, this approach can sometimes lead to upper body ischemia. Recent studies have suggested using VA-ECMO support through the axillary artery and femoral vein, as this may offer antegrade perfusion and help prevent cerebral ischemia (Fig. 1).

## Conclusion

Hypoxemia is a significant complication that can arise after surgery for ATAAD. Several potential risk factors contribute to the development of hypoxemia, including the patient’s smoking history, obesity, inflammatory response before and after surgery, advanced age, preoperative renal insufficiency, duration of CBP and DHAC, and intraoperative blood product transfusion. Hypoxemia not only prolongs the patient’s need for mechanical ventilation and ICU treatment, but also diminishes the survival rate. Therefore, for patients with ATAAD who have risk factors for postoperative hypoxemia, comprehensive preoperative evaluation and perioperative management are essential. By implementing appropriate intervention measures, the occurrence of postoperative hypoxemia can be minimized and prevented.

## Data Availability

No datasets were generated or analysed during the current study.

## Abbreviations

ATAAD: Acute type A aortic dissection
DHCA: Deep hypothermic circulatory arrest
HO: Hypoxemia
CPB: Cardiopulmonary bypass
PaO_2_: Pressure of arterial oxygen
TRALI: Transfusion-related acute lung injury
ICU: Intensive Care Unit
PP: Prone position
ARDS: Acute respiratory distress syndrome
EELV: End-expiratory lung volume
FRC: Functional residual capacity
HFNO: High-flow nasal oxygen therapy
NO: Nitric oxide
LPS: Lipopolysaccharide
UTI: Ulinastatin
ECMO: Extracorporeal membrane oxygenation
